# Increased rates of chronic physical health conditions across all organ systems in autistic adolescents and adults

**DOI:** 10.1186/s13229-023-00565-2

**Published:** 2023-09-20

**Authors:** John H. Ward, Elizabeth Weir, Carrie Allison, Simon Baron-Cohen

**Affiliations:** 1 Royal Devon University NHS Foundation Trust, Exeter, Devon, UK; 2 University of Exeter Medical School, Devon, UK; 3 Autism Research Centre, Department of Psychiatry, University of Cambridge, Douglas House, 18b Trumpington Road, CB2 8AH, Cambridge, UK; 4 University of Oxford, Department of Psychiatry, Oxford, UK; 5 Oxford Health NHS Foundation Trust, Oxford, UK

**Keywords:** Autism, Physical health, Chronic illness, Ehlers-Danlos syndrome, Gastrointestinal condition, Neurological condition, Rheumatological condition

## Abstract

**Background:**

The poorer physical health of autistic adults compared to non-autistic adults has been highlighted by several epidemiological studies. However, research has so far been limited to specific geographical areas and has primarily focused on young autistic individuals (aged 35 years and younger). Recent studies indicate a higher rate of mortality in autistic people, as well as poorer quality of self-reported healthcare interactions. This study aims to determine, first, whether autistic people experience greater levels of non-communicable health conditions and second, whether these are explained by differences in demographics (i.e. sex, country of residence, ethnicity, education level), alcohol use, smoking, body mass index (BMI), or family history of medical conditions.

**Method:**

We employed a cross-sectional, convenience-sampling study via an anonymous, online survey of autistic and non-autistic adults (n = 2305, mean age = 41.6, 65.9% female, 49% autistic). The survey asked participants to self-report information about their demographics, autism diagnosis, diet, exercise, sleep, sexual health, substance use, personal medical history, and family medical history (for all first-degree, biological relatives). Binomial logistic regression across four iterative models of increasing complexity was applied to assess rates of physical health conditions. The Benjamini–Hochberg correction was used to account for multiple testing, and only physical health conditions that achieved at least 1% endorsement within the overall sample (n > 22) were included in the analysis to reduce risk of Type I errors. We also used novel network analysis methods to test whether there are increased levels of multimorbidity between autistic and non-autistic people.

**Results:**

There were significantly elevated rates of non-communicable conditions across all organ systems in autistic people, including gastrointestinal, neurological, endocrine, visual, ear/nose/throat, skin, liver and kidney, and haematological conditions. We confirmed previous findings by showing highly significant differences in rates of neurological and gastrointestinal symptoms (*p* < 0.0001). In addition, we established in the largest sample to date that Ehler-Danlos Syndrome (EDS) was more likely to occur among autistic females compared to non-autistic females. Finally, we found a higher prevalence of Coeliac’s disease among autistic individuals compared to non-autistic individuals after controlling for sex, ethnicity, country of residence, alcohol use, smoking, and BMI, but these results became non-significant after accounting for family history.

**Limitations:**

Our study is biased towards females, white individuals, highly educated people, and UK residents, likely due to sampling biases. Our self-report study design may also exclude those who lack access to computers, or those with intellectual disability. Our network analysis is also limited in size.

**Conclusions:**

This study provides evidence of widespread, physical health comorbidity that spans nearly all major organ systems in autistic adults compared to non-autistic adults, using both binary logistic regression and network models. Healthcare professionals must be made aware of the range of co-occurring physical health conditions that may be more common among autistic people. However, our findings also point towards potential avenues requiring further exploration, such as the association of autism with both Coeliac’s disease and EDS.

**Supplementary Information:**

The online version contains supplementary material available at 10.1186/s13229-023-00565-2.

## Background

Autism spectrum conditions (henceforth autism) are a heterogenous set of neurodevelopmental conditions involving differences in social communication alongside strong interests, and unusually repetitive behaviours that present from early childhood. Current estimates suggest that 2.8% of children are now diagnosed as autistic by the age of eight years old [1]. Autistic individuals have increased risks of co-occurring physical and mental health conditions, and have significantly increased risk of mortality and reduction in life expectancy. This may be partly attributable to deaths from neurological causes (including epilepsy/seizures), cancers, and suicide, although these do not fully account for this concerning disparity in lifespan [2–8].

The data examining the health gap in autism are currently limited, both in size and scope [9]. As is shown in Table 1, the majority of studies have utilised population-based data from the United States to examine the health of autistic adolescents and young adults [9–11], although one study employed Medicare data from adults aged 65 years and older [12]. A few cohort studies also show higher rates of both physical health conditions and unmet healthcare needs [13, 14] among autistic people. Rydzewska et al. [15] used Scottish census data to illustrate that autistic individuals are 2.6 times more likely (95% CI 2.5–2.8, *p* < 0.001) to have a physical health condition compared to their non-autistic counterparts. However, whilst this study has a very large sample size (n > 3.7 million, including 6649 autistic people) and permits stratification of analyses by age range, it does not provide any additional data on the type of condition, or the number of conditions that affect the autistic population. Furthermore, a recent self-report study also showed increased rates of cardiovascular, respiratory, and diabetic conditions among autistic people [16]. Existing research is limited by the lack of breadth of conditions or organ systems, as well as a limited number of studies addressing risk of multimorbidity (i.e., a person having physical health conditions that affect multiple organ systems, such as diabetes and Ehlers-Danlos Syndrome, EDS) [9, 17]. In addition, the above studies largely focus on a relatively young age range and include low numbers of autistic females (likely due to underdiagnosis) [18, 19]. Finally, many of the studies use a case–control design and depend on pre-existing data (i.e., coded clinical data). Whilst coded clinical data has clear advantages of no recall bias, clinical oversight of diagnoses and comprehensiveness over time, it has several limitations [12, 20]. These include lack of precision regarding individual lifestyle factors (e.g., body mass index (BMI), smoking, alcohol use) or family history, and missing data; this type of data precludes recording of diagnoses that have not been formally coded (e.g., sensory issues without formal diagnosis).

**Table 1:** Studies to date examining incidence of physical health disorders in autistic versus non-autistic people

Paper	Sample size	Data source/method	Findings
Bishop-Fitzpatrick et al. [38]	91 autistic, 6186 non-autistic	Machine learning paradigm constructed from electronic health records, ICD-9 codes, V-codes and E-codes	Higher rates of cardiovascular, motor, ear, urinary and digestive problems in autistic people, as well as long-term medication side effects. Lower rates of cancer in autistic people
Davignon et al. [20]	4123 autistic, 20,615 with ADHD, 2156 with diabetes, 20,615 non-autistic	Health records of 14–25 year olds from Kaiser Permanente Northern California (KPNC)	Elevated rates of nearly all organ systems studied (developmental delay, psychiatric disorder, allergies, cardiovascular, endocrine, ear/nose/throat, gastrointestinal, genetic, haematology/oncology, injuries, metabolic, musculoskeletal, neurological, nutritional, overweight/obesity, ophthalmological, pulmonary, sleep and smoking) in autism versus typical controls
Fortuna et al. [16]	255 autistic adults	Rochester Health Status Survey IV was used to collect data on health status from 18–79 year olds. The results were compared with national prevalence rates	Higher rates of seizure disorder in the 18–29 year olds and over 40 year olds studied than the general population. 18–29 year olds more likely to have hypertension and allergies
Hand et al. [12]	4685 autistic, 46,850 non-autistic	Comparison of medicare data amongst older adults (65 +)	Higher rates of epilepsy, Parkinson’s disease and gastrointestinal disorders. Also higher rates of osteoporosis, cognitive disorders, heart disease, cancer, cerebrovascular disease and osteoarthritis
Hirvikoski et al. [2]	27,122 autistic, 2,672,185 non-autistic	Case cohort study on data from Swedish National Patient Register and Cause of Death Register	Odds ratio of 2.56 for mortality in autistic versus non-autistic people during the studied period. Mean age at death 70.2 among non-autistic people versus 53.9 in autistic people
Kohane et al. [9]	14,381 autistic versus 2,379,397 non-autistic	Retrospective prevalence study using ICD-9 hospital data from 18–34 year olds	Higher rates of epilepsy, gastrointestinal disorders and neurological disorders in autistic versus non-autistic people
Croen et al. [11]	1507 autistic, 15,070 non-autistic	Kaiser Permanente Northern California data, using ICD-9 codes of adults over 18	Higher rates of gastrointestinal disorders, neurological disorders (including strokes and Parkinson’s Disease), sleep disorders, seizures, obesity, dyslipidaemia, hypertension and diabetes
Rydzewska et al. [15]	6649 autistic versus 3,739,935 non-autistic	Scottish Census Data for over 25 year olds	Higher rates of sensory impairments, intellectual disability and physical disability in autistic versus non-autistic people
Tint et al. [39]	6870 autistic women versus 2,686,160 non-autistic women	Reproductive age women (15–44) studied using administrate health data, using ICD and DSM codes	Significantly higher rates of diabetes and teratogenic medication use, and higher primary care use
Turcotte and Shea [76]	1197 autistic people	Pennsylvania autism needs assessment study data (a self-report survey) of over 18 year olds	Higher use of physical health services amongst autistic people
Vohra et al. [71]	1772 autistic versus 5320 non-autistic	Medicaid data for 22–64 year olds in Illinois, New York and Texas	Higher rates of epilepsy, skin disorders and hearing impairments. Also more outpatient appointments, prescription drug use and higher health expenditure overall
Weiss et al. [40]	5095 autistic versus 10,487 with other developmental disorders versus 393,263 typical controls	Health administrative data from 18 to 24 year olds in Ontario, Canada	Higher rates of diabetes, hypertension, asthma and substance use disorders in autistic versus non-autistic people. Additionally, greater number of visits to hospital specialists
Weir et al. *[31]	1156 autistic versus 1230 non-autistic	Self-report data of diagnosed non-communicable illnesses via survey from adults, regardless of physical health/mental health status	Higher rates of cardiovascular/respiratory conditions, as well as diabetes, even when controlling for demographic and lifestyle related factors

*Weir et al. [31] draws upon the same dataset as this present paper, however it should be stressed that the analyses presented in both papers are mutually-exclusive

Based on the current literature, there does not appear to be one pathophysiological mechanism that describes the increased health burden among autistic adults. Whilst genes may play a role, given the heritability of autism [21], there is also a growing body of research demonstrating poorer quality healthcare interactions and self-reported healthcare experience for autistic people [22–25], as well as structural barriers in healthcare like the mainstreaming of telehealth post-pandemic [26]. There are also significant disparities in the social determinants of health (e.g. employment, socioeconomic status, exposure to trauma) which likely influence the health of autistic people [27–30].

The present study aims to compare the long-term physical health of autistic and non-autistic adults, taking into account a broad range of demographic variables (e.g., sex assigned at birth, ethnicity, education-level, age, and country of residence) and dynamic risk factors for health outcomes (e.g., body mass index (BMI), smoking, and alcohol use). In addition, the study aims to examine the interrelationships between chronic physical health conditions among autistic versus non-autistic people. Whilst this group has previously published work on the prevalence of cardiovascular conditions, respiratory conditions, diabetes and cancer prevalence using the same dataset as the present paper, the aim of this paper is to investigate the lifetime prevalence of numerous other medical conditions self-reported by autistic and non-autistic participants, and examine the interactions between these conditions [31].

## Methods

### Study design

This study compared rates of physical and mental health conditions among autistic and non-autistic adolescents and adults via an online, anonymized, and cross-sectional survey (conducted via Qualtrics) using a convenience sampling design.

### Participants

There were 3657 responses to the survey. Anyone aged 16 years or older, with or without an autism diagnosis, was eligible for inclusion in the study. Participants were recruited via databases such as the Cambridge Autism Research Database (CARD) and Autistica’s Discover Network, social media, and via autism charities and support groups. The survey was only available in English. Data were collected between February 2018 and August 2019, with two pauses in recruitment. A sensitivity analysis for all analyses confirmed that there was no undue influence of recruitment phase on the results. The results of this sensitivity analysis are included in Additional file 1: Table S1. There were several exclusion criteria for the study. Participants who suspected that they were autistic but not diagnosed, were awaiting diagnosis, or were self-diagnosed as autistic were excluded from this study (n = 33). As the study was anonymised, we developed an algorithm to exclude suspected duplicate responses. Responses were removed if they included identical answers to a previous response across 13 criteria. These factors were: autism diagnosis (including precise diagnosis and by whom it was diagnosed), year of diagnosis, known genetic mutations, maternal and paternal age at birth, participant’s age, country of residence, assigned sex at birth, gender identity, highest level of educational qualification, and Autistic Spectrum Quotient (AQ) score. Accordingly, n = 87 participants were excluded from the analysis.

Finally, our models could only include people who had complete family and medical histories. This led to the exclusion of 1232 responses of individuals who did not complete the survey in full. Further information on these participants can be found in Additional file 1. After all exclusions, the final number of participants in the study was n = 2305 (including 1129 autistic participants).

### Questionnaire

For the purposes of this study, we created a new questionnaire (the Autism and Physical Health Survey, APHS) with multiple branching sections (see Additional file 2). The first section of the questionnaire enquired about demographics (sex assigned at birth, age, country of residence, ethnicity, education level) and autism diagnosis. Only participants who stated that they had received a diagnosis of autism by a qualified professional were included in this study. While autism diagnosis was self-reported, participants were required to provide the specific diagnosis received, type of practitioner that diagnosed them, the year in which they were diagnosed, and whether they were diagnosed with any syndromic forms of autism. These details were used to confirm a clinical diagnosis made by a professional; additionally, participants who disclosed that they were awaiting a diagnosis, suspected they were autistic, or were self-diagnosed were excluded from our analyses.

With respect to physical health, the questionnaire asked about height and weight [used to calculate body mass index (BMI)], smoking status, and alcohol consumption, and recreational drug use. Participants were also asked to disclose whether they have ever had (i.e. over the whole lifespan) any physical health conditions that fell within a particular body system, with examples provided; participants were able to select multiple categories. These categories were *cancer* (with sex-specific breakdown); *heart condition; risk of stroke; lung condition; digestive/gastrointestinal condition; hormone/endocrine condition* (with sex-specific breakdown); *reproductive condition; muscle and bone/musculoskeletal condition; neurological condition; eye condition; ear, nose, and throat condition; liver or kidney condition; blood or lymph condition; skin condition; diabetes or prediabetes; autoimmune condition*; or none of the above. If participants selected a category (e.g., *heart condition*), they were invited to select and/or specify (via free text box) what condition[s] they had. Psychiatric conditions were not included in this study as this was designed as a study of chronic physical health conditions. Additionally, transient conditions without long-term consequence (e.g. infections) and spurious acquired illnesses (e.g., traumatic injuries) were also excluded from analysis. Findings for risk of cardiovascular conditions, respiratory conditions, diabetes, and cancer have been reported elsewhere [31], and are therefore not reported in regression analyses here. They have however, been included in our novel application of network analysis (apart from our cancer findings due to cancer not being an organ system disorder).

The physical health conditions listed in the survey were identified by accessing publicly available material from the National Health Service, World Health Organisation, Cancer Research UK, National Institutes of Health (NIH), National Institute for Health and Care Excellence. We discussed our final list of conditions with clinicians to ensure clinical relevance and good coverage of common physical health conditions among the general population.

#### Family history

Participants were asked to disclose medical history data about all first-degree, biological relatives [who share approximately 50% of their deoxyribonucleic acid (DNA)], including their mother and father, as well as up to five of each of the following: brothers, sisters, sons, and daughters. For each family member selected, participants were then asked if they had an autism diagnosis or any medical conditions, using a similar drop-down. Responses were then coded as binary variables, both at the resolution of the organ system affected (e.g., family history of a gastrointestinal condition) and at the level of the condition (e.g., family history of Coeliac’s Disease).

### Statistical analysis

Statistical analyses were performed using *R* Version 4.1.2. Data were manually cleaned using the *stringr* and *grep* packages to standardise participant responses (e.g., standardizing spellings and units). Demographic variables were compared between groups using chi-squared tests for category membership (e.g., sex assigned at birth) and Wilcoxon tests for continuous variables, performed within the *CrossTable* package. The results of these analyses are in Table 3.

The primary analyses utilized binomial logistic regressions, using the *glm function* within the *stats* package of *R*. Four, iterative binomial logistic regression models were used (Table 2). Covariates were coded in the following ways: sex assigned at birth (binary), ethnicity (binary: white vs. non-white), age (continuous), country of residence (categorical: United Kingdom (UK), United States of America (USA), and Other), Education (categorical: no formal education to high school qualification, undergraduate-level qualification, and postgraduate-level qualification), family history (binary), BMI (continuous), smoking status (binary: daily vs. non-daily smoking), and alcohol consumption (categorical: no alcohol, 1–2 alcoholic beverages per average session, 3–4, 5–6, 7–8, or more than 8).

**Table 2:** Summary of model structure used in this analysis

Model 1	Model 2	Model 3	Model 4
Autism	Autism	Autism	Autism
	Sex assigned at birth	Sex assigned at birth	Sex assigned at birth
	Ethnicity	Ethnicity	Ethnicity
	Country of residence	Country of residence	Country of Residence
	Age	Age	Age
	Education level	Education level	Education level
		Family history	Smoking
			Alcohol
			BMI

A multiple comparison correction was applied to reduce Type I errors using the Benjamini–Hochberg methods, with a false discovery rate set at 5% [32]. Models were calculated firstly for each organ system (e.g., gastrointestinal, neurological) and then calculated again for all discrete conditions with at least a 1% prevalence across the sample (e.g., inflammatory bowel disease, migraine) to mitigate risk of performing underpowered analyses. Where appropriate and clinically relevant, rarer conditions were aggregated into larger categories so that they could be analysed (e.g., tendinopathies in different anatomical locations, seizure types).

One advantage of using a self-report design is that there was less than 3% missingness for any individual variable. Multiple imputation was used via the multiple imputation chained equations (MICE) package in *R* to preserve our sample size. We used five imputations of our dataset using predictive mean matching to impute missing values for covariates in our data. We did not use multiple imputation for autism status or for any outcomes. Adjusted models were then calculated using an interpolation of the five imputations of each participant’s data. The results were then pooled according to Rubin’s Rules [33]. Some adjusted models presented issues with perfect separation with respect to the covariate sex assigned at birth (specifically for male participants). Where this has happened, we have presented only the female models, as models of male participants were uninterpretable due to relatively smaller numbers of males in the sample; we also included only female models for relevant sex-specific conditions. Thus, rates of the following conditions were only reported for females: polycystic ovary syndrome, premenstrual tension, endometriosis, fibroids, hypermobility, and Ehler-Danlos Syndrome.

Network analysis was used to compare the relatedness of conditions across different organ system between autistic and non-autistic participants. Network analysis was conducted using the Ising Model, developed by van Borkulo et al. [34] to study networks in binary datasets [34]. The *psychonetrics* package in *R* was used in order to apply a false discovery rate (FDR) threshold to the findings to reduce the effects of multiple testing [35]. *NetworkComparisonTest* was used to compare the global strength, edge weights and node centralities between networks [36]. The network comparison test was applied using 1000 iterations.

## Results

The sample included 2305 participants (comprising 1129 autistic and 1176 non-autistic participants). The mean age of autistic participants 40.9 years (standard deviation (SD) 14.5 years) and non-autistic participants was 41.6 years (SD 15.5) with an age range from 16 to 90 years. The sample was biased toward individuals assigned female at birth, white participants, residents of the United Kingdom, individuals who completed a University-level educational qualification or higher, overweight/obese individuals, and those who do not drink or smoke. Between our autistic and non-autistic groups, non-autistic individuals were significantly more likely to be assigned female at birth, to drink alcohol, to have lower BMI on average, to have a relatively higher education-level on average, and to reside in the United States, Ireland, or ‘Other’ countries. Descriptive analyses and between-group comparisons are presented in Table 3.

**Table 3:** Table demonstrating the demographics within our sample between groups, with P values reported

Characteristic	Autistic (n = 1129)	Non-autistic (n = 1176)	Statistical significance
Mean age (SD)	40.9 (14.5)	41.6 (15.5)	0.39
Age categories, N (%)			
Under 29	297 (26.31)	310 (26.36)	
30–39	237 (20.99)	237 (20.15)	
40–49	243 (21.52)	240 (20.41)	
50–59	204 (18.07)	210 (17.86)	
60–69	103 (9.12)	118 (10.03)	
70 +	26 (2.30)	49 (4.17)	
Missing	19 (1.68)	12 (1.02)	
Sex assigned at birth N (%)			0.01
Male	413 (36.58)	372 (31.63)	
Female	716 (63.42)	804 (68.37)	
Missing	0	0	
Ethnicity, N (%)			0.02
White	994 (88.04)	999 (84.95)	
Mixed race	76 (6.73)	68 (5.78)	
Asian	17 (1.51)	43 (3.66)	
Latin/Hispanic	6 (0.53)	22 (1.87)	
Arab/Middle Eastern	0	17 (1.45)	
Jewish	17 (1.51)	17 (1.45)	
Black	6 (0.53)	9 (0.77)	
OTHER	10 (0.89)	1 (0.09)	
Missing	3 (0.27)	0	
Education			< 0.0001
No Formal qualifications	52 (4.61)	16 (1.36)	
Further vocational qual	207 (18.33)	137 (11.65)	
Secondary/High School	205 (18.16)	169 (14.37)	
University (undergraduate)	337 (29.85)	346 (29.42)	
University (postgraduate)	326 (28.88)	507 (43.11)	
Missing	2 (0.18)	1 (0.09)	
Country of residence, N (%)			< 0.0001
UK	802 (71.04)	739 (62.84)	
USA	113 (10.01)	171 (14.54)	
Germany	30 (2.66)	33 (2.81)	
Ireland	13 (1.15)	27 (2.30)	
Canada	23 (2.04)	22 (1.87)	
Australia	31 (2.75)	21 (1.79)	
Netherlands	29 (2.57)	8 (0.68)	
Other	88 (7.79)	151 (12.84)	
Missing	1 (0.09)	4 (0.34)	
BMI, mean (SD)	27.1 (7.62)	25.9 (6.17)	9.3 × 10^–4^
Missing	34 (3.01)	34 (2.89)	
Daily smoker N (%)			0.63
No	790 (69.97)	812 (69.05)	
Yes	338 (29.94)	363 (30.87)	
Missing	1 (0.09)	1 (0.09)	
Current alcohol frequency, N (%)			< 0.0001
0	658 (58.28)	459 (39.03)	
1–2	291 (25.78)	457 (38.86)	
3–5	114 (10.10)	177 (15.05)	
6–7	65 (5.76)	83 (7.06)	
Missing	1 (0.09)	0 (0)	

Chi-Squared for percentage differences and t-test for mean differences

Conditions in all organ systems were more common in autistic people versus non-autistic people, even after accounting for age, sex assigned at birth, ethnicity, country of residence, education-level, BMI, smoking, alcohol use, and family history. Differences were significant at the organ system-level for all organ systems tested below the *p* < *0.0001* threshold, except for haematological and endocrine conditions (which were significant at the *p *< 0.05 level). In particular, our results suggest that autistic people were two to three times more likely to have gastrointestinal, rheumatological, neurological, and renal/hepatic conditions. Unadjusted and adjusted estimates for the risk of having at least one condition in each organ system between autistic versus non-autistic individuals are provided in Table 4.

**Table 4:** Table denoting the odds ratios (ORs) with 95% confidence intervals and false discovery rate (FDR) adjusted *p* values for our four tiers of model; unadjusted, demographic adjusted, family history adjusted and lifestyle adjusted

	Non-autistic participants (%)	Autistic participants (%)	Model 1		Model 2		Model 3		Model 4	
			Odds ratio	FDR	Odds ratio	FDR	Odds ratio	FDR	Odds ratio	FDR
			(95% CI)	*p* value	(95% CI)	*p* value	(95% CI)	*p* value	(95% CI)	*p* value
**Gastrointestinal**	20.25	38.56	2.47	***p* < 0.0001**	2.83	***p* < 0.0001**	2.75	***p* < 0.0001**	2.69	***p* < 0.0001**
			(2.05–2.98)		(2.26–3.55)		(2.18–3.46)		(2.14–3.39)	
Irritable bowel syndrome	11.75	25.00	2.50	***p* < 0.0001**	2.57	***p* < 0.0001**	2.44	***p* < 0.0001**	2.44	***p* < 0.0001**
			(2.01–3.14)		(1.99–3.33)		(1.88–3.18)		(1.87–3.17)	
Ulcerative colitis (M)	1.88	0.48	0.25	0.11	0.26	0.12	0.26	0.13	0.26	0.14
			(0.04–1.06)		(0.05–1.31)		(0.05–1.32)		(0.05–1.39)	
Ulcerative colitis (F)	1.12	1.96	1.76	0.21	1.70	0.26	1.73	0.24	1.77	0.74
			(0.77–4.25)		(0.71–4.07)		(0.72–4.17)		(0.72–4.35)	
Coeliac Disease	0.85	2.13	2.53	**0.02**	2.76	**0.0193**	2.17	0.09	2.55	**0.0364**
			(1.24–5.57)		(1.24–6.17)		(0.95–4.96)		(1.13–5.76)	
Faecal incontinence	1.11	3.72	3.46	***p* < 0.0001**						
			(1.90–6.73)							
Gastric reflux	8.43	19.77	2.68	***p* < 0.0001**	2.74	***p* < 0.0001**	2.80	***p* < 0.0001**	2.60	***p* < 0.0001**
			(2.09–3.46)		(2.03–3.69)		(2.06–3.81)		(1.92–3.53)	
Peptic ulcers	1.11	1.95	1.78	0.13	1.81	0.17	1.90	0.14	1.72	0.21
			(0.90–3.64)		(0.82–4.03)		(0.85–4.25)		(0.77–3.86)	
Hernia (any)	1.19	5.14	4.50	***p* < 0.0001**	4.54	**1.0 × 10**^**–4**^	4.38	**1.8 × 10**^**–4**^	4.54	**1.4 × 10**^**–4**^
			(2.57–8.43)		(2.22–9.28)		(2.14–8.98)		(2.2–9.36)	
Gastroparesis	0.43	2.22	5.30	**1.4 × 10**^**–3**^						
			(2.20–15.80)							
Gallbladder disease	0.68	2.22	3.31	**5.9 × 10**^**–3**^	3.18	**0.02**	3.09	**0.02**	2.95	**0.04**
			(1.55–7.86)		(1.27–7.95)		(1.24–7.72)		(1.15–7.61)	
Gallstones	1.96	3.99	2.08	**7.7 × 10**^**–3**^	2.40	**6.0 × 10**^**–3**^	2.40	**6.5 × 10**^**–3**^	1.95	**0.05**
			(1.26–3.52)		(1.33–4.33)		(1.33–4.36)		(1.06–3.58)	
Diverticulosis	0.85	1.51	1.78	0.19	2.32	0.11	2.30	0.13	2.29	0.13
			(0.83–4.05)		(0.87–6.16)		(0.86–6.19)		(0.84–6.26)	
Chronic diarrhoea	1.87	7.80	4.43	***p* < 0.0001**	3.92	***p* < 0.0001**	3.87	***p* < 0.0001**	3.71	***p* < 0.0001**
			(2.81–7.30)		(2.30–6.68)		(2.26–6.63)		(2.17–6.36)	
Chronic constipation	3.75	10.11	2.89	***p* < 0.0001**	3.22	***p* < 0.0001**	3.22	***p* < 0.0001**	2.96	***p* < 0.0001**
			(2.04–4.17)		(2.13–4.88)		(2.10–4.94)		(1.94–4.51)	
Functional pain	0.94	3.55	3.89	**2.1 × 10**^**–4**^						
			(2.06–8.00)							
Haemorrhoids	4.77	9.66	2.14	***p* < 0.0001**	2.20	**3.4 × 10**^**–4**^	2.16	**6.2 × 10**^**–4**^	2.06	**1.4 × 10**^**–3**^
			(1.54–3.00)		(1.47–3.31)		(1.43–3.27)		(1.36–3.11)	
GI polyps	1.70	3.64	2.18	**7.7 × 10**^**–3**^	2.29	**0.02**	2.23	***p* < 0.0001**	2.33	**0.0201**
			(1.28–3.81)		(1.19–4.42)		(1.15–4.32)		(1.20–4.53)	
**Endocrine**	17.02	20.57	1.56	**0.04**	1.54	**8.8 × 10**^**–4**^	1.31	**0.01**	1.48	**3.8 × 10**^**–3**^
			(1.26–1.56)		(1.21–1.96)		(1.10–1.81)		(1.15–1.89)	
Polycystic ovarian syndrome (F)	5.23	8.81	1.75	**0.01**	1.85	**5.8 × 10**^**–3**^	1.89	**5.0 × 10**^**–3**^	1.59	**0.05**
			(1.17–2.64)		(1.22–2.80)		(1.25–2.88)		(1.04–2.43)	
Premenstrual tension (F)	8.59	13.99	1.73	**1.8 × 10**^**–3**^	1.89	**4.3 × 10**^**–3**^	1.54	**0.02**	1.81	**1.6 × 10**^**–3**^
			(1.25–2.40)		(1.35–2.64)		(1.09–2.18)		(1.28–2.55)	
Endometriosis (F)	3.74	6.57	1.81	**0.02**	2.09	**4.8 × 10**^**–3**^	1.98	**0.01**	1.89	**0.02**
			(1.14–2.93)		(1.29–3.39)		(1.21–3.23)		(1.15–3.09)	
Fibroids (F)	3.61	2.79	0.77	0.40	0.92	0.78	0.63	0.19	0.81	0.54
			(0.43–1.36)		(0.50–1.67)		(0.33–1.22)		(0.44–1.50)	
Precocious puberty	0.60	1.77	2.01	**0.02**						
			(1.33–7.70)							
Hypothyroidism	6.13	6.11	1.00	0.99	1.28	0.22	1.11	0.61	1.17	0.45
			(0.71–1.40)		(0.89–1.84)		(0.76–1.62)		(0.80–1.71)	
Hyperthyroidism	1.45	1.41	0.98	0.97						
			(0.49–1.96)							
**Rheumatological**	14.72	25.44	1.98	***p* < 0.0001**	2.33	***p* < 0.0001**	2.2	***p* < 0.0001**	2.16	***p* < 0.0001**
			(1.60–2.44)		(1.81–3.01)		(1.69–2.87)		(1.66–2.80)	
Rheumatoid arthritis	2.30	3.28	1.44	0.19	1.27	0.48	1.18	0.63	1.31	0.45
			(0.88–2.41)		(0.68–2.39)		(0.63–2.23)		(0.69–2.48)	
Osteoarthritis	6.81	8.16	1.22	0.24	1.44	0.09	1.43	0.13	1.39	0.14
			(0.89–1.66)		(0.96–2.16)		(0.93–2.20)		(0.92–2.09)	
Carpal tunnel syndrome	1.87	5.14	2.84	**1.2 × 10**^**–4**^	3.53	***p* < 0.0001**	3.83	***p* < 0.0001**	3.46	***p* < 0.0001**
			(1.75–4.77)		(2.01–6.21)		(2.13–6.89)		(1.95–6.13)	
Scoliosis	2.21	3.90	1.79	**0.03**	1.65	0.09	1.54	0.16	1.61	0.21
			(1.11–2.97)		(0.95–2.87)		(0.87–2.72)		(0.92–2.82)	
Spinal stenosis	1.91	1.24	1.04	0.94	1.81	0.26	1.87	0.24	1.61	0.13
			(0.49–2.21)		(0.67–4.89)		(0.68–5.13)		(0.92–2.82)	
Slipped disc	1.79	4.97	2.87	**1.4 × 10**^**–4**^	3.34	**6.1 × 10**^**–4**^	2.84	**4.5 × 10**^**–3**^	3.09	**1.8 × 10**^**–3**^
			(1.75–5.88)		(1.74–6.42)		(1.45–5.56)		(1.59–6.01)	
Fibromyalgia	1.79	6.65	3.91	***p* < 0.0001**	3.95	***p* < 0.0001**	3.69	***p* < 0.0001**	3.49	***p* < 0.0001**
			(2.44–6.55)		(2.37–6.68)		(2.18–6.26)		(2.04–5.95)	
Hypermobility (F)	0.62	4.48	7.48	**1.1 × 10**^**–4**^	7.47	**1.1 × 10**^**–4**^	5.62	**1.2 × 10**^**–3**^	6.05	**6.7 × 10**^**–4**^
			(3.17–21.97)		(2.87–19.44)		(2.11–15.00)		(2.30–15.9)	
Ehler-Danlos syndrome (F)	0.87	6.29	7.64	***p* < 0.0001**	7.74	***p* < 0.0001**	6.81	***p* < 0.0001**	7.34	***p* < 0.0001**
			(3.65–18.65)		(3.44–17.42)		(2.97–15.60)		(3.23–16.65)	
**Neurological**	17.87	29.70	1.94	***p* < 0.0001**	2.24	***p* < 0.0001**	2.16	***p* < 0.0001**	2.14	***p* < 0.0001**
			(1.60–2.36)		(1.77–2.83)		(1.71–2.73)		(1.68–2.71)	
Migraine	14.81	22.70	1.69	***p* < 0.0001**	1.81	***p* < 0.0001**	1.76	***p* < 0.0001**	1.74	***p* < 0.0001**
			(1.37–2.09)		(1.41–2.31)		(1.36–2.28)		(1.35–2.24)	
Epilepsy (M)	2.96	1.94	0.65	0.39	0.68	0.47	0.67	0.42	0.69	0.50
			(0.25–1.62)		(0.25–1.80)		(0.25-1.78)		(0.25–1.90)	
Epilepsy (F)	1.12	5.18	4.81	**1.0 × 10**^**–4**^	5.09	***p* < 0.0001**	4.65	**2.1 × 10**^**–4**^	4.69	**2.0 × 10**^**–4**^
			(2.41–10.69)		(2.40–10.78)		(2.18–9.90)		(2.20–10.02)	
Syncope	1.02	3.64	3.66	**2.3 × 10**^**–4**^	4.19	**2.1 × 10**^**–4**^	4.00	**3.9 × 10**^**–4**^	4.00	**5.0 × 10**^**–4**^
			(1.97–7.30)		(2.05–8.57)		(1.95–8.21)		(1.94–8.27)	
Vertigo	1.28	3.64	2.92	**9.5 × 10**^**–4**^	4.00	**2.5 × 10**^**–4**^	4.33	**2.1 × 10**^**–4**^	3.73	**7.0 × 10**^**–4**^
			(1.64–5.47)		(1.99–8.05)		(2.10–8.93)		(1.83–7.58)	
Chronic Fatigue syndrome	2.21	6.56	3.10	***p* < 0.0001**	3.33	***p* < 0.0001**	3.17	***p* < 0.0001**	3.19	***p* < 0.0001**
			(2.00–4.97)		(2.03–5.50)		(1.92–5.24)		(1.92–5.29)	
**Ocular**	35.83	44.24	1.42	**1.2 × 10**^**–4**^	1.63	***p* < 0.0001**	1.54	**3.9 × 10**^**–4**^	1.61	***p* < 0.0001**
			(1.20–1.68)		(1.31–2.01)		(1.23–1.93)		(1.29–2.00)	
Nearsightedness	28.40	31.10	1.14	0.19	1.26	0.06	1.19	0.19	1.24	0.07
			(0.95–1.36)		(1.01–1.58)		(0.93–1.51)		(0.98–1.56)	
Farsightedness	8.51	11.35	1.38	**0.03**	1.32	0.11	1.26	0.20	1.32	0.13
			(1.05–1.82)		(0.96–1.82)		(0.90–1.76)		(0.95–1.83)	
Astigmatism	18.30	25.10	1.50	**2.2 × 10**^**–4**^	1.76	***p* < 0.0001**	1.70	**1.2 × 10**^**–4**^	1.75	***p* < 0.0001**
			(1.22–1.83)		(1.38–2.25)		(1.32–2.19)		(1.36–2.25)	
Blurred vision	1.53	4.43	2.98	**2.3 × 10**^**–4**^	3.19	**1.2 × 10**^**–3**^	3.00	**1.9 × 10**^**–3**^	2.93	**2.8 × 10**^**–4**^
			(1.76–5.28)		(1.62–5.95)		1.56–5.76)		(1.52–5.66)	
Diplopia	0.77	2.75	3.66	**1.3 × 10**^**–3**^						
			(1.81–8.20)							
Colourblindness	1.19	1.60	1.34	0.43	3.53	0.08	3.37	0.08	2.98	0.14
			(0.67–2.76)		(0.93–13.4)		(0.88–12.96)		(0.77–11.5)	
Strabismus	0.94	2.57	2.79	**6.7 × 10**^**–3**^						
			(1.43–5.87)							
Amblyopia	3.15	6.29	2.07	**9.7 × 10**^**–3**^	2.11	**5.1 × 10**^**–3**^	2.03	**8.8 × 10**^**–3**^	2.01	**0.01**
			(1.39–3.13)		(1.29–3.47)		(1.23–3.35)		(1.21–3.34)	
Cataract	3.83	3.46	0.90	0.66	0.87	0.67	0.88	0.69	0.92	0.79
			(0.58–1.39)		(0.46–1.63)		(0.47–1.66)		(0.48–1.74)	
Blindness	1.87	2.75	1.48	0.19	2.3	0.07	2.23	0.08	2.06	0.12
			(0.86–2.60)		(1–5.28)		(0.97–5.14)		(0.89–4.80)	
Otolaryngology	9.70	17.73	2.01	***p* < 0.0001**	1.96	***p* < 0.0001**	1.82	**2.1 × 10**^**–4**^	1.87	**1.2 × 10**^**–4**^
			(1.57–2.57)		(1.46–2.62)		(1.35–2.45)		(1.39–2.51)	
Deafness	1.02	2.93	2.92	**2.9 × 10**^**–3**^	2.18	0.07	2.02	0.11	2.1	0.09
			(1.54–5.92)		(0.99–4.79)		(0.92–4.47)		(0.95–4.68)	
Tinnitus	2.13	7.89	3.94	***p* < 0.0001**	3.91	***p* < 0.0001**	3.81	***p* < 0.0001**	3.81	***p* < 0.0001**
			(2.55–6.31)		(2.26–6.76)		(2.19–6.62)		(2.19–6.63)	
Mouth ulcers	2.55	5.76	2.33	**3.6 × 10**^**–4**^	2.18	**3.6 × 10**^**–3**^	2.11	**5.9 × 10**^**–3**^	2.06	**8.7 × 10**^**–3**^
			(1.52–3.67)		(1.33–3.57)		(1.29–3.48)		(1.24–3.40)	
TMJ syndrome	1.11	3.72	3.46	**2.5 × 10**^**–4**^	4.06	**1.2 × 10**^**–3**^	3.63	**9.8 × 10**^**–4**^	3.62	**5.8 × 10**^**–4**^
			(1.90–6.73)		(2.07–7.94)		(1.77–7.46)		(1.83–7.14)	
**Renal/hepatic**	3.75	8.33	2.34	***p* < 0.0001**	2.48	**1.3 × 10**^**–4**^	2.32	**5.2 × 10**^**–4**^	2.21	**1.1 × 10**^**–3**^
			(1.63–3.40)		(1.60–3.84)		(1.49–3.62)		(1.41–3.46)	
Non-alcoholic fatty liver disease	0.51	1.51	2.98	**0.03**						
			(1.23–8.29)							
Kidney stones	1.02	1.68	1.66	0.20	1.36	0.54	1.28	0.63	1.19	0.74
			(0.81–3.53)		(0.53–3.49)		(0.49–3.30)		(0.45–3.1)	
Urinary incontinence	1.02	3.99	4.03	***p* < 0.0001**	5.20	***p* < 0.0001**	5.32	***p* < 0.0001**	4.57	**1.7 × 10**^**–4**^
			(2.19–8.00)		(2.53–10.70)		(2.54–11.20)		(2.18–9.54)	
**Haematological**	7.40	11.34	1.60	**2.3 × 10**^**–3**^	1.75	**8.6 × 10**^**–4**^	1.67	**3.1 × 10**^**–3**^	1.76	**2.1 × 10**^**–3**^
			(1.21–2.14)		(1.28–2.39)		(1.21–2.29)		(1.28–2.41)	
Iron deficiency anaemia	5.79	8.78	1.57	**9.3 × 10**^**–3**^	1.74	**2.8 × 10**^**–3**^	1.69	**5.8 × 10**^**–3**^	1.74	**3.6 × 10**^**–3**^
			(1.14–2.16)		(1.24–2.45)		(1.19–2.4)		(1.22–2.47)	
**Dermatological**	21.78	29.97	1.54	***p* < 0.0001**	1.67	***p* < 0.0001**	1.70	***p* < 0.0001**	1.65	***p* < 0.0001**
			(1.27–1.85)		(1.33–2.09)		(1.35–2.14)		(1.32–2.08)	
Eczema	16.25	21.28	1.39	**3.7 × 10**^**–3**^	1.50	**2.4 × 10**^**–3**^	1.56	**1.3 × 10**^**–3**^	1.48	**4.0 × 10**^**–3**^
			(1.13–1.72)		(1.17–1.92)		(1.21–2.01)		(1.15–1.90)	
Psoriasis	3.75	7.54	2.09	**2.5 × 10**^**–5**^	1.76	**0.02**	1.76	**0.02**	1.70	0.06
			(1.45–3.07)		(1.12–2.76)		(1.11–2.79)		(1.07–2.68)	
Acne	7.40	13.03	1.87	***p* < 0.0001**	2.18	***p* < 0.0001**	2.09	***p* < 0.0001**	2.19	***p* < 0.0001**
			(1.42–2.48)		(1.57–3.04)		(1.48–2.95)		(1.57–3.07)	
Hyperhidrosis	0.51	1.86	3.70	**7.8 × 10**^**–3**^						
			(1.58–10.10)							
Rosacea	0.68	1.24	1.83	0.20						
			(0.78–4.61)							

Values in bold are at the *p *=  < 0.05 level

Furthermore, when examining specific physical health conditions, both unadjusted and adjusted models (accounting for demographics, alcohol use, smoking, BMI, and family history) suggest that autistic individuals are more likely to have specific physical health conditions than non-autistic individuals for the vast majority of conditions tested. Differences in prevalence of specific physical health conditions are widespread across all organ systems, although there are particularly large group differences in prevalence rates of neurological and rheumatological conditions (see Table 3).

Several conditions remained significant after accounting for our demographic variables, alcohol use, smoking, BMI, and family history of the condition. These include: irritable bowel syndrome (IBS), gastric reflux, GI tract hernias, gallbladder disease, gallstones, chronic diarrhoea, chronic constipation, haemorrhoids, GI polyps, polycystic ovarian syndrome (females only), premenstrual tension (females only), endometriosis (females only), carpal tunnel syndrome, slipped discs, hypermobility (females only), Ehler-Danlos Syndrome (EDS) (females only), migraine, epilepsy (females only), syncope, vertigo, chronic fatigue syndrome, astigmatism, blurred vision, amblyopia, tinnitus, mouth ulcers, temporomandibular joint syndrome, urinary incontinence, iron deficiency anaemia, eczema, psoriasis, and acne. Autistic people had significantly higher risk of coeliac’s disease when adjusting for demographics, alcohol use, smoking, and BMI, but this finding became non-significant  after accounting for family history.

We also performed an age stratification, dividing our sample around our mean age (under 41, 41 and over) (see Table 5) This demonstrated higher levels of disease burden across nearly all organ systems among both younger autistic people and older autistic people (compared to their respective non-autistic, age-stratified peers), aligning with our overall results presented in Table 3. The only notable difference was that levels of endocrine disorder in those over the age of 40 was similar amongst our autistic and non-autistic populations.

**Table 5:** Age stratified analysis of the likelihood of disorders of different organ systems, in a (top; 40 and under) and b (40 and over)

	Non-autistic participants (%)	Autistic participants (%)	Model 1		Model 2		Model 3		Model 4	
			Odds ratio	FDR	Odds ratio	FDR	Odds ratio	FDR	Odds ratio	FDR
				*p* value		*p* value		*p* value		*p* value
*(a) Under 41*										
Gastro	16.67	36.15	2.83	< 0.0001	3.20	< 0.0001	3.15	< 0.0001	3.26	< 0.0001
Neurological	16.66	26.26	1.92	< 0.0001	1.95	< 0.0001	1.86	< 0.0001	1.95	< 0.0001
Endocrine	14.63	21.40	1.59	0.0046	1.75	0.0029	1.65	0.0098	1.78	0.0030
Rheumatological	6.54	20.14	3.60	< 0.0001	3.89	< 0.0001	3.67	< 0.0001	3.83	< 0.0001
ENT	8.61	16.91	2.16	< 0.0001	2.21	4.1 × 10^–4^	2.12	0.0011	2.10	0.0013
Ocular	31.67	39.59	1.41	0.0062	1.61	0.003	1.43	0.038	1.53	0.0087
Renal/hepatic	2.75	6.30	2.37	0.0062	2.39	0.020	2.33	0.030	–	–
Haematological	7.40	10.97	1.54	0.038	1.47	0.091	1.34	0.21	1.40	0.15
Dermatological	21.69	29.68	1.52	0.0038	1.58	0.0060	1.58	0.0098	1.56	0.0087
*(b) 41 and over*										
Gastro	23.37	41.41	2.31	*P *< 0.0001	2.35	*P *< 0.0001	2.24	*P *< 0.0001	2.18	*P *< 0.0001
Neurological	20.28	33.64	1.99	*P *< 0.0001	2.30	*P *< 0.0001	2.24	*P *< 0.0001	2.18	*P *< 0.0001
Endocrine	19.07	19.89	1.05	0.73	1.26	0.20	1.14	0.49	1.21	0.32
Rheumatological	23.20	31.28	1.51	0.0029	1.70	0.0056	1.60	0.010	1.63	0.0097
ENT	10.65	18.63	1.92	5.0 × 10^–4^	1.63	0.029	1.49	0.080	1.52	0.066
Ocular	40.03	50.09	1.50	0.0010	1.58	0.0056	1.58	0.011	1.61	0.0029
Renal/hepatic	4.81	10.49	2.32	7.5 × 10^–4^	2.33	0.0056	2.18	0.012	2.14	0.013
Haematological	7.39	12.12	1.73	0.0086	1.84	0.0095	1.82	0.013	1.97	0.0086

### Network analysis

**Fig. 1:**
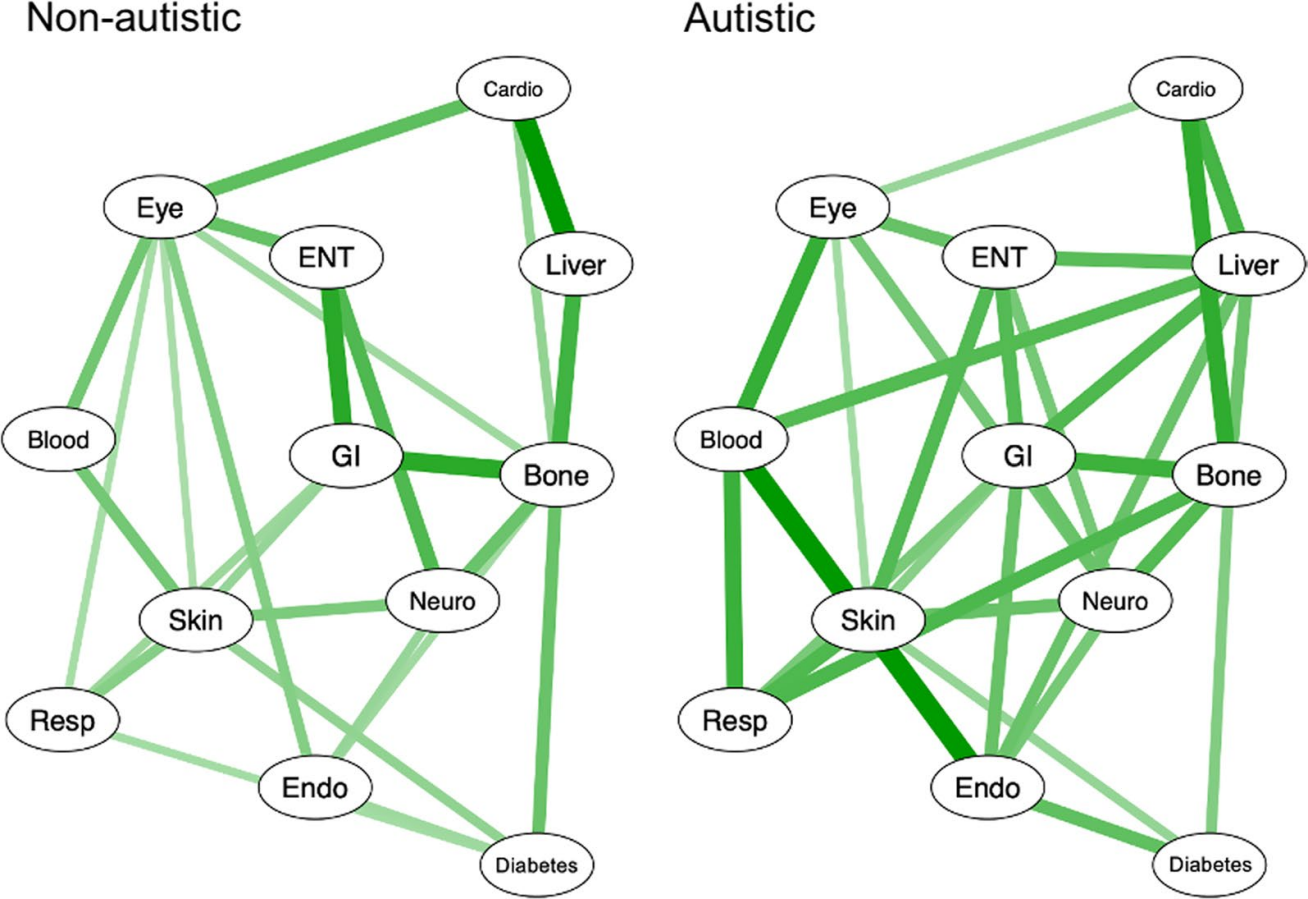
Ising Model network analysis of reporting of conditions by organ system seen in the non-autistic (L) versus autistic (R) sample. Multiple comparison correction applied

The network models are presented in Fig. 1. There are a greater number of connections in the autistic sample and the network is more densely packed, denoted by the greater number and greater thickness of lines. The Network Comparison Test demonstrated a difference in the overall global network strength (*p* = 0.032), indicating a greater number of edges between nodes in the autistic versus non-autistic sample.

## Discussion

The results of this study suggest that autistic people are at greater risk of a breadth of chronic physical health conditions, as well as overall disease burden across the lifespan, without an observable pattern, alluding to a combination of complex biological and environmental interactions that produce a widespread, increased physical health burden among autistic adults.

Given the above, there are findings of interest which warrant further investigation. In particular, our results suggest that autistic people may have elevated rates of rheumatological conditions, consistent with what was found by Davignon et al. [20]. Ehlers-Danlos Syndrome (EDS) was frequently reported in females in our sample, despite not being provided as an explicit option within our original survey (with participants instead using the provided free text box). The strength of the association in our sample was such even when controlling for family history, autism was still significantly associated with EDS (despite EDS being a genetic condition). A link between autism and EDS has previously been suggested through analysis of co-implicated genes (e.g., *matrix metalloproteinase 9*) but there has not previously been such a strong association in a sample of this scale [37]. Although our results appear to indicate group differences in rates of EDS, this should be confirmed using large, population-based samples, adopting both epidemiological and genomic approaches (to identify genes co-implicated in autism and connective tissue disorders), as the present results could also be accounted for by sampling biases. In addition, sex-specific rates should be further analysed.

Our age-stratified analyses provide evidence that both younger and older autistic adults experience elevated rates of conditions across nearly all organ systems compared to similarly aged non-autistic adults–aligning with findings from the majority of other studies on this topic [2, 9, 11, 12, 20, 38–40]. Interestingly, the prevalence of endocrine disorders were similar in the over 41 age group, independent of autism, despite endocrine disorders being significantly elevated among younger autistic adults (compared to younger non-autistic adults). Whilst this may relate to biases of our sample, it may also relate to time trends in endocrine diagnoses over time, for example PCOS and endometriosis have become more readily recognized and diagnosed over the last decade [41, 42].

Further research is also needed to understand the high rates of medically unexplained symptoms and central sensitivity syndromes reported by autistic individuals in this study. Medically unexplained symptoms are defined as symptoms without a known organic cause (e.g., chronic diarrhoea that is not caused by irritable bowel disease or a known infection, tinnitus without structural pathology). Overall, whilst there is some research considering some particular medically unexplained symptoms in autism, there is a lack of research considering medically unexplained symptoms as a whole among autistic people [43, 44]. Further research examining how these diagnoses are reached in autistic people versus non autistic people would be useful, to understand whether this diagnosis is applied the same irrespective of autism or whether the increase in medically unexplained symptoms in autistic people is actually a symptom of the known structural and communication barriers encountered by autistic people in healthcare [23–25, 45].

Central sensitivity syndromes (CSSs) are a heterogeneous group of conditions which primarily cause pain that is thought to be due to the sensitisation of the central nervous system [46, 47]. CSSs may include migraine, tinnitus, Temporomandibular Joint Syndrome (TMJ), and fibromyalgia. A recent study of 973 people in the Netherlands found that 21% of autistic adults had a CSS diagnosis, and 60% had sufficient symptoms to warrant a CSS diagnosis [48]. However, their results must be taken with caution as there was no control group in this study. The current study found elevated rates of these same CSS conditions among autistic compared to non-autistic people, specifically in relation to TMJ syndrome, Irritable Bowel Syndrome (IBS), Myalgic Encephalomyelitis/Chronic Fatigue Syndrome (ME/CFS) and Fibromyalgia. Our study thus goes further in finding increased rates of CSSs in autistic versus non-autistic people.

Furthermore, the results from our network analysis suggest that the inter-relation of chronic health conditions to each other is different between autistic and non-autistic people, both in the strength and structure of relationships. The difficulties in the translational application of network analysis have been previously explored, with consensus that statistics should be interpreted cautiously and in context of what nodes represent [49–51]. The conservative interpretation of our network analysis therefore is that it provides preliminary evidence of physical health multi-morbidity in autistic people, and that perhaps there may be different patterns of multi-morbidity of these conditions than those seen in the non-autistic population. However, this requires examining in much larger samples, to improve interpretability of particular clusters of interest. Furthermore, given that our study asked about health conditions across the lifespan (i.e. conditions participants had ever had, and not specifically excluding conditions diagnosed in childhood), it is important to further investigate physical health multimorbidity by examining the temporal associations of diagnoses, including the temporal associations of these medical events to autism diagnoses. The latter may be informative in understanding how different diagnoses are applied in autistic versus non-autistic people, as discussed above. Furthermore, examining more contemporaneous medical history may minimise the influence of recall bias in recollecting medical events over the course of one’s lifetime. Following Brunson et al. [49], we organised conditions by organ system in our analysis, which may introduce some artefacts in those conditions with ramifications across different organ systems (e.g., syncope, where the cause of collapse could be theoretically cardiac or neurological). An alternative approach in future analyses (which applies beyond network analysis) is that the data could analysed by pathophysiological mechanism. This may be of particular relevance when assessing rates of certain conditions among autistic vs. non-autistic people, as there may be mechanisms related to genetic conditions or central sensitivity syndromes.

Whilst our results are largely congruent with the findings from previous studies, there are some important differences. First, our results found no difference when examining the rates of epilepsy/seizures between autistic and non-autistic males (although not for females). This is at odds with previous research which demonstrates higher rates of epilepsy in autistic versus non-autistic people [12, 52]. The most likely explanation of this finding is that epilepsy/seizures are most prevalent among autistic individuals with co-occurring intellectual disability (ID) (a previous estimate has suggested an incidence of 214 per 1000 in autism/intellectual disability versus 80 per thousand in autistic people without ID) [53, 54]. Due to the sampling method employed, our sample is biased toward autistic females and autistic individuals without co-occurring ID. Thus it is likely that the current sample is not well-suited to assessing risk of epilepsy/seizures among autistic males. It is understood from previous meta-analytic evidence that autistic women are more at risk of epilepsy than their male counterparts, although our results cannot speak to this given our gender bias [55].

Either way, this is a limitation of using self-report as a methodology to conduct research of this nature. This highlights that these results should be viewed as preliminary and that they must be examined in larger, population-based samples. Second, there were some other differences (e.g., lower rates of blindness, inflammatory bowel disease, and hypothyroidism in our study versus others, and higher rates of migraine among autistic participants) that also differed from what has been previously been reported; as above, these findings may be a result of under-sampling of individuals with co-occurring ID or male participants, or of other sampling biases [11, 15, 16, 56].

This study includes the largest sample to date of autistic individuals across the lifespan on the topic of self-reported health status and co-occurring physical health conditions (including large numbers of middle-aged and older autistic adults). In addition, it includes a large number of autistic females, who remain underserved in autism research. Third, another advantage of our self-report methodology was very low levels of missingness within our dataset (< 3% for any variable). This is an important strength given our large sample size, and given the high rates of missingness that can be encountered when analysing administrative or medical data originally collected for routine medical purposes [57]. Fourth, the study utilizes a far wider range of covariates than has been previously employed, including four levels of model adjustment incorporating covariates for age, ethnicity, sex assigned at birth, country of residence, education-level, alcohol use, smoking, BMI, and family history of medical conditions. This robust analysis strategy allows us to further assess the contributing factors that may or may not impact upon the rates of chronic physical health conditions for autistic compared to non-autistic individuals. Finally, this study has covered a more specific range of physical health conditions than any previous studies in this area, allowing finer grain analysis of the challenges faced by autistic people regarding physical health burden. Overall, the study uses a different methodology to come to similar conclusions as many other studies [11, 12, 15, 20]. While the results from this study should not be taken alone, they build upon a larger literature that collectively indicate broadly higher risk of physical health conditions among autistic people compared to others.

Whilst we did not undertake patient public involvement (PPI) for this study, we recognise the importance of PPI in relating research findings to the day-to-day lives of autistic people. We feel strongly that future work in the physical health of autistic adults should incorporate the views of autistic people and autistic researchers [58, 59].

## Limitations

First, our method, as exemplified by our non-significant findings regarding epilepsy/seizures among autistic males, may be subject to sampling biases that may lead to over- or under-estimations regarding rates of physical health conditions among autistic individuals. Further, both our autistic and non-autistic samples are biased towards white individuals, those assigned female at birth, residents of the United Kingdom, have a University-level education or higher, those who are overweight/obese, and those who do not drink or smoke. Our study is also biased towards female participants, as is expected for self-report survey studies [60, 61]. Thus, these results may be limited in their applicability to other groups and are unlikely to be representative of all autistic or non-autistic people. Considering recently published research suggesting that autism may be more prevalent among non-white (and particularly Black) individuals, it is urgent that more research is conducted on the rates of co-occurring physical and mental health conditions among individuals with diverse ethnic backgrounds [62]. It should be noted that only 21% of the non-autistic sample and 13% of the autistic sample reported no physical health conditions in the categories examined, which may provide further evidence of sampling biases. Furthermore, we recognise that our use of self-report methodology means that some people may have endorsed health diagnoses for which they have not received formal assessment (but have become aware of through social media channels and online fora). Additionally, our sampling method may have also excluded those without internet access, those who are unable to complete a lengthy survey, and those with a learning disability/intellectual disability that limits their ability to participate in this type of research. Given the known associations between intellectual disability and physical health, this should be further examined in the context of autism [63]. Second, we have also excluded from our samples those who have not been formally diagnosed with autism. Theoretically, it is possible (and perhaps inherently likely) that individuals who have not yet received diagnoses may also have poorer access to physical health services. Future research should focus on identifying the prevalence of co-occurring physical and mental health risks among individuals who are awaiting an autism diagnosis.

Third, there are also limitations to our network analysis. The Ising Model is still in development and has not been previously applied in this context. Using this model to compare samples with different sizes should be done with caution [36]. However, we have used FDR correction to try to account for false positives and used 1000 permutations of our network comparison test to help account further for these.

Fourth, a limitation of our study is our definition and measurement of ‘lifestyle’ variables. There are nuances around alcohol and smoking (e.g. intensity, frequency, periods of ‘bingeing’) which may impact on health but cannot be easily measured, as has been previously demonstrated by clinical research into the sensitivity of history taking [64, 65]. Future work, perhaps focussing around conditions with robust association with social behaviour (e.g. alcohol and smoking-related disorders), may be better suited to explore lifestyle factors in greater resolution than we have in our work.

We note that the social determinants of health are much broader than one’s ‘health behaviours’, including a myriad of factors that would be important when considering autistic people, such as stigma, educational attainment, employment outcomes and mental health needs, all of which may impact on one’s health as an adult [27–29, 66, 67]. Whilst our study’s self-report methodology would not lend itself to quanitifying social determinants of health across different geographic contexts, we note the importance better capturing this important factor in the health of minority communities.

## Conclusion

Our results suggest that there are greater risks of co-occurring physical health conditions and complex health needs across the lifespan among autistic people compared to non-autistic people. This is supported by several previous papers using a wide range of methodologies and samples [11, 12, 15, 20]. This may be due to biological contributors (e.g., genetic or hormonal) to risk of these physical health conditions and/or due to social/economic issues related to negative life experiences, stigma, as well as poorer self-reported healthcare quality [23, 24, 37, 68–72]. Importantly, no singular cause of the poorer physical health of autistic emerges from the existing literature or the present study. [24, 37]. Whilst there is growing support for more specific healthcare channels for autistic people, including an annual health check in the United Kingdom, at the very simplest level we recommend that healthcare professionals should be vigilant for signs and symptoms of physical illness among autistic people, and should be providing reasonable adjustments to care to improve healthcare interactions [23, 73–75].

### Supplementary Information

**Additional file 1: Table S1.** Table demonstrating the demographics of the excluded 309 participants with sufficient data for analysis.

**Additional file 2:** Questions 59–71 of physical health survey, denoting the physical health questions explicitly asked of participants.

## Data Availability

Data are available on application for collaboration to the Autism Research Centre.

## Abbreviations

AQ: Autism spectrum quotient
CARD: Cambridge autism research database
CSS: Central sensitivity syndrome
CTS: Carpal tunnel syndrome
DNA: Deoxyribonucleic acid
EDS: Ehlers-Danlos syndrome
FDR: False discovery rate
IBS: Irritable bowel syndrome
ID: Intellectual disability
ME/CFS: Myalgic encephalomyelitis/chronic fatigue syndrome
MICE: Multiple imputation by chained equations
TMJ: Temporomandibular joint [syndrome]
UK: United Kingdom
USA: United States of America
