# Supplementary material for: Increased rates of chronic physical health conditions across all organ systems in autistic adolescents and adults
Source: Mol Autism. 2023 Sep 20;14:35. doi: 10.1186/s13229-023-00565-2 (PMC10510241; doi:10.1186/s13229-023-00565-2)
Supplement: Supplementary file 1 — Additional file 1: Table S1. Table demonstrating the demographics of the excluded 309 participants with sufficient data for analysis. [file 13229_2023_565_MOESM1_ESM.docx]

**Supplementary material**

**Excluded Participant Analysis**

As stated in the main body of the paper, 1,232 participants were excluded for having incomplete medical or family histories. 914 of these responses lacked complete demographic information (and many did not consent to participation). As such, we are unable to provide further information on the demographics of these individuals. Of the 318 for which there was some information available, 8 responses among the remaining group proved to be duplicate responses and were excluded and 1 further intersex individual was excluded (due to perfect separation issues). The final group of individuals excluded for incomplete response included 309 individuals. Analysis indicated that this group is highly similar to the study’s sample across all demographics. Full details on their demographics have been reported in Supplementary Table 1.

| Characteristic | Autistic (n=164) | Control (n=145) |
| --- | --- | --- |
| Mean Age (SD) | 40.12 (14.81) | 40.43 (16.11) |
| Age Categories, N (%) |  |  |
| Under 29 | 46 (28.05) | 28 (19.31) |
| 30-39 | 36 (21.95) | 30 (20.69) |
| 40-49 | 30 (18.29) | 37 (25.52) |
| 50-59 | 33 (20.12) | 19 (13.10) |
| 60-69 | 16 (9.76) | 18 (12.41) |
| 70+ | 1 (0.61) | 11 (7.59) |
| Missing | 2 (1.22) | 2 (1.38) |
|  |  |  |
| Sex assigned at birth N (%) |  |  |
| Male | 62 (37.80) | 51 (35.17) |
| Female | 102 (62.20) | 94 (64.83) |
| Missing | 0 | 0 |
|  |  |  |
| Ethnicity, N (%) |  |  |
| White | 144 (87.80) | 117 (80.69) |
| Mixed Race | 9 (5.49) | 13 (8.97) |
| Asian | 1 (0.61) | 5 (3.45) |
| Latin/Hispanic | 1 (0.61) | 4 (2.76) |
| Arab/Middle Eastern | 1 (0.61) | 3 (2.07) |
| Jewish | 3 (1.83) | 3 (2.07) |
| Black | 2 (1.22) | 0 |
| Other | 2 (1.22) | 0 |
| Missing | 1 (0.61) | 0 |
|  |  |  |
| Education |  |  |
| No Formal Qualifications | 7 (4.27) | 2 (1.22) |
| Further vocational qual | 36 (21.95) | 18 (12.41) |
| Secondary/High School | 32 (19.51) | 25 (17.24) |
| University (undergraduate) | 42 (25.61) | 41 (28.28) |
| University (postgraduate) | 47 (28.66) | 57 (39.31) |
| Missing | 0 | 2 (1.38) |
|  |  |  |
| Country of Residence, N (%) |  |  |
| UK | 127 (77.44) | 91 (62.76) |
| USA | 17 (10.37) | 14 (9.66) |
| Germany | 2 (1.22) | 4 (2.76) |
| Ireland | 0 | 6 (4.14) |
| Canada | 1 (0.61) | 5 (3.45) |
| Australia | 2 (1.22) | 1 (0.69) |
| Netherlands | 2 (1.22) | 1 (0.69) |
| Other | 0 | 0 |
| Missing | 0 | 0 |
|  |  |  |
| BMI, mean (SD) | 27.29 (6.94) | 26.73 (6.66) |
| Missing | 7 | 1 |
|  |  |  |
| Daily Smoker N (%) |  |  |
| No | 81 (49.39) | 65 (44.83) |
| Yes | 35 (21.34) | 48 (33.10) |
| Missing | 48 (29.27) | 32 (22.07) |
|  |  |  |
| Current Alcohol Frequency, N (%) |  |  |
| 0 | 74 (45.12) | 46 (31.72) |
| 1-2 | 22 (13.41) | 46 (31.72) |
| 3-5 | 12 (7.34) | 19 (13.10) |
| 6-7 | 9 (5.49) | 4 (2.76) |
| Missing | 47 (28.66) | 30 (20.69) |

Supplementary Table 1: table demonstrating the demographics of the excluded 309 participants with sufficient data for analysis.
