# Supplementary material for: Increased rates of chronic physical health conditions across all organ systems in autistic adolescents and adults
Source: Mol Autism. 2023 Sep 20;14:35. doi: 10.1186/s13229-023-00565-2 (PMC10510241; doi:10.1186/s13229-023-00565-2)
Supplement: Supplementary file 2 — Additional file 2: Questions 59–71 of physical health survey, denoting the physical health questions explicitly asked of participants. [file 13229_2023_565_MOESM2_ESM.docx]

**Supplementary Material: questions 59-71 of physical health survey, denoting the physical health questions explicitly asked of participants.**

Q59 Which of the following conditions have you ever had? Please select all that apply:

- Irritable Bowel Syndrome (IBS) (1)
- Crohn's Disease (2)
- Ulcerative Colitis (3)
- Celiac Disease (4)
- Bowel Control Problems (Fecal Incontinence) (5)
- Acid Reflux (6)
- Peptic Ulcer Disease (7)
- Cyclic Vomiting Syndrome (8)
- Gastroparesis (9)
- Hernia (10)
- Pancreatitis (11)
- Gallbladder Disease (12)
- Gallstones (13)
- Diverticular Disease (14)
- Chronic Diarrhea (15)
- Chronic Constipation (16)
- Abdominal Migraine/ Functional Abdominal Pain (17)
- Hemorrhoids (18)
- Hirschsprung Disease (19)
- Polyps (20)
- Other Digestive Condition (Please Specify): (21) ________________________________________________

| Page Break |  |
| --- | --- |

Display This Question:

If Have you ever been told that you have any of the following medical conditions? Please select all... = Reproductive Condition (for example: early-onset puberty or premenstrual syndrome)

Or Have you ever been told that you have any of the following medical conditions? Please select all... = Hormone/ Endocrine Condition (for example: growth hormone or thyroid conditions)

And If

Which gender was assigned to you at birth? != Male

Q60 Which of the following conditions have you ever had? Please select all that apply:

- Anovulation (1)
- Congenital Adrenal Hyperplasia (CAH) (2)
- Polycystic Ovarian Syndrome (PCOS) (3)
- Premenstrual Syndrome (PMS) (4)
- Endometriosis (5)
- Uterine Prolapse (6)
- Hypogonadism/ Kallmann Syndrome (7)
- Precocious/ Early-Onset Puberty (8)
- Delayed Puberty (9)
- Prolactinoma (10)
- Craniopharyngioma (11)
- Fibroids (12)
- Cushing's Disease (13)
- Goiter (14)
- Overactive Thyroid/ Hyperthyroidism (15)
- Underactive Thyroid/ Hypothyroidism (16)
- Overactive Parathyoid/ Hyperparathyroidism (17)
- Underactive Parathyroid/ Hypoparathyroidism (18)
- Hypersecretion of Growth Hormone/ Acromegaly (19)
- Hyposecretion of Growth Hormone/ Growth Hormone Deficiency (20)
- Other Hormonal or Reproductive Condition (Please Specify): (21) ________________________________________________

Display This Question:

If Have you ever been told that you have any of the following medical conditions? Please select all... = Reproductive Condition (for example: early-onset puberty or premenstrual syndrome)

Or Have you ever been told that you have any of the following medical conditions? Please select all... = Hormone/ Endocrine Condition (for example: growth hormone or thyroid conditions)

And If

Which gender was assigned to you at birth? != Female

Q61 Which of the following conditions have you ever had? Please select all that apply:

- Congenital Adrenal Hyperplasia (CAH) (1)
- Hypogonadism/ Kallmann Syndrome (2)
- Precocious/ Early-Onset Puberty (3)
- Delayed Puberty (4)
- Prolactinoma (5)
- Craniopharyngioma (6)
- Cushing's Disease (7)
- Goiter (8)
- Overactive Thyroid/ Hyperthyroidism (9)
- Underactive Thyroid/ Hypothyroidism (10)
- Overactive Parathyroid/ Hyperparathyroidism (11)
- Underactive Parathyroid/ Hypoparathyroidism (12)
- Hypersecretion of Growth Hormone/ Acromegaly (13)
- Hyposecretion of Growth Hormone/ Growth Hormone Deficiency (14)
- Other Hormonal or Reproductive Condition (Please Specify): (15) ________________________________________________

| Page Break |  |
| --- | --- |

Display This Question:

If Have you ever been told that you have any of the following medical conditions? Please select all... = Muscle and Bone/ Musculoskeletal Condition (for example: arthritis)

Q62 Which of the following conditions have you ever had? Please select all that apply:

- Rheumatoid Arthritis (1)
- Osteoarthritis (2)
- Carpal Tunnel Syndrome (3)
- Scoliosis (4)
- Spinal Stenosis (5)
- Slipped Disc (6)
- Spina Bifida (7)
- Osteoporosis (8)
- Brittle Bone Disease (9)
- Paget's Disease (10)
- Muscular Dystrophy (11)
- Lupus (12)
- Fibromyalgia (13)
- Scleroderma (14)
- Other Muscle or Bone Condition (Please Specify): (15) ________________________________________________

| Page Break |  |
| --- | --- |

Display This Question:

If Have you ever been told that you have any of the following medical conditions? Please select all... = Neurological Condition (for example: migraine or syncope)

Q63 Which of the following conditions have you ever had? Please select all that apply:

- Dementia (including Alzheimer's Disease) (1)
- Migraine (2)
- Cerebral Palsy (3)
- Multiple Sclerosis (4)
- Motor Neuron Disease (including ALS) (5)
- Parkinson's Disease (6)
- Epilepsy/ Seizure Disorder (7)
- Frequent Fainting/ Syncope (8)
- Recurring Vertigo (9)
- Autoimmune Encephalitis (10)
- Chronic Fatigue Syndrome (CFS)/ Myalgic Encephalomyelitis (ME)/ Post-Viral Fatigue Syndrome (PVFS) (11)
- Other Neurological Condition (Please Specify): (12) ________________________________________________

| Page Break |  |
| --- | --- |

Display This Question:

If Have you ever been told that you have any of the following medical conditions? Please select all... = Eye Condition (for example: cataracts or nearsightedness)

Q64 Which of the following conditions have you ever had? Please select all that apply:

- Nearsightedness (1)
- Farsightedness (2)
- Astigmatism (3)
- Frequent or Persistent Blurred Vision (4)
- Frequent or Persistent Double vision/ Diplopia (5)
- Color Blindness (6)
- Crossed Eyes/ Strabismus (7)
- Lazy Eye/ Amblyopic (8)
- Cataracts (9)
- Diabetic Retinopathy (10)
- Glaucoma (11)
- Macular Degeneration (12)
- Retinal Detachment (13)
- Full or Partial Sight Loss/ Blindness (14)
- Other Eye Condition (Please Specify): (15) ________________________________________________

| Page Break |  |
| --- | --- |

Display This Question:

If Have you ever been told that you have any of the following medical conditions? Please select all... = Ear, Nose, or Throat Condition (for example: frequent mouth ulcers or Sjögren's syndrome)

Q65 Which of the following conditions have you ever had? Please select all that apply:

- Hearing Impaired/ Deaf (1)
- Ménière's disease (2)
- Tinnitus (3)
- Frequent Mouth Ulcers (4)
- Sjögren's syndrome (5)
- TMJ Disorders (6)
- Anosmia/ Absent or Persistently Reduced Sense of Smell (7)
- Dysphagia (8)
- Other Ear, Nose, and Throat Condition (Please Specify): (9) ________________________________________________

| Page Break |  |
| --- | --- |

Display This Question:

If Have you ever been told that you have any of the following medical conditions? Please select all... = Liver or Kidney Condition (for example: urinary incontinence)

Q66 Which of the following conditions have you ever had? Please select all that apply:

- Alcohol-Related Liver Disease (1)
- Non-Alcoholic Liver Disease (2)
- Primary Biliary Cholangitis (3)
- Chronic Kidney Disease (4)
- Kidney Stones (5)
- Acute Kidney Injury (6)
- Urinary Incontinence (7)
- Diabetes Insipidus (8)
- Other Liver or Kidney Condition (Please Specify): (9) ________________________________________________

| Page Break |  |
| --- | --- |

Display This Question:

If Have you ever been told that you have any of the following medical conditions? Please select all... = Blood or Lymph Condition (for example: anemia)

Q67 Which of the following conditions have you ever had? Please select all that apply:

- Sickle Cell Anemia (1)
- Cooley's Anemia (2)
- Iron Deficiency Anemia (3)
- Hemochromatosis (4)
- Hemophilia (5)
- von Willebrand Disease (6)
- Deep Vein Thrombosis (DVT) (7)
- Hyperkalemia (8)
- Lymphoedema (9)
- Castleman Disease (10)
- Other Blood or Lymph Condition (Please Specify): (11) ________________________________________________

| Page Break |  |
| --- | --- |

Display This Question:

If Have you ever been told that you have any of the following medical conditions? Please select all... = Skin condition (for example: eczema or hyperhidrosis)

Q68 Which of the following conditions have you ever had? Please select all that apply:

- Eczema (1)
- Psoriasis (2)
- Acne (3)
- Hidradenitis Suppurativa (4)
- Pressure Ulcer (5)
- Albinism (6)
- Vitiligo (7)
- Alopecia Areata (8)
- Hyperhidrosis (9)
- Other Skin Condition (Please Specify): (10) ________________________________________________

| Page Break |  |
| --- | --- |

Q70 Please list all autoimmune conditions that you have, even if listed above:

________________________________________________________________

________________________________________________________________

________________________________________________________________

________________________________________________________________

________________________________________________________________

| Page Break |  |
| --- | --- |

Q71 Is there anything else that you think would be important to disclose about your health or medical history?

________________________________________________________________

________________________________________________________________

________________________________________________________________

________________________________________________________________

________________________________________________________________
